# 6FDA-DAM:DABA Co-Polyimide Mixed Matrix Membranes with GO and ZIF-8 Mixtures for Effective CO_2_/CH_4_ Separation

**DOI:** 10.3390/nano11030668

**Published:** 2021-03-08

**Authors:** Anand Jain, Mohd Zamidi Ahmad, Audrey Linkès, Violeta Martin-Gil, Roberto Castro-Muñoz, Pavel Izak, Zdeněk Sofer, Werner Hintz, Vlastimil Fila

**Affiliations:** 1 Department of Inorganic Technology, University of Chemistry and Technology Prague, Technická 5, 166 28 Prague 6, Czech Republic; ady.jain92@gmail.com (A.J.); audrey.linkes@gmail.com (A.L.); violeta.m.gil@gmail.com (V.M.-G.); or castromr@tec.mx (R.C.-M.); 2 Faculty of Process and Systems Engineering, Otto-von-Guericke-University, Magdeburg Universitätsplatz 2, 39106 Magdeburg, Germany; werner.hintz@ovgu.de; 3 Organic Materials Innovation Center (OMIC), Department of Chemistry, University of Manchester, Oxford Road, Manchester M13 9PL, UK; 4 Tecnologico de Monterrey, Campus Toluca, Avenida Eduardo Monroy Cárdenas 2000 San Antonio Buenavista, Toluca de Lerdo 50110, Mexico; 5 Department of Membrane Separation Processes, Institute of Chemical Process Fundamentals of the CAS, v. v. i., Rozvojova 2/135, 165 02 Prague 6, Czech Republic; izak@icpf.cas.cz; 6 Department of Inorganic Chemistry, University of Chemistry and Technology Prague, Technická 5, 166 28 Prague 6, Czech Republic; Zdenek.Sofer@vscht.cz

**Keywords:** 6FDA-polyimide, mixed matrix membranes, ZIF-8/GO, CO_2_ separation

## Abstract

This work presents the gas separation evaluation of 6FDA-DAM:DABA (3:1) co-polyimide and its enhanced mixed matrix membranes (MMMs) with graphene oxide (GO) and ZIF-8 (particle size of <40 nm). The 6FDA-copolyimide was obtained through two-stage poly-condensation polymerization, while the ZIF-8 nanoparticles were synthesized using the dry and wet method. The MMMs were preliminarily prepared with 1–4 wt.% GO and 5–15 wt.% ZIF-8 filler loading independently. Based on the best performing GO MMM, the study proceeded with making MMMs based on the mixtures of GO and ZIF-8 with a fixed 1 wt.% GO content (related to the polymer matrix) and varied ZIF-8 loadings. All the materials were characterized thoroughly using TGA, FTIR, XRD, and FESEM. The gas separation was measured with 50:50 vol.% CO_2_:CH_4_ binary mixture at 2 bar feed pressure and 25 °C. The pristine 6FDA-copolyimide showed CO_2_ permeability (*P*_CO2_) of 147 Barrer and CO_2_/CH_4_ selectivity (*α*_CO2/CH4_) of 47.5. At the optimum GO loading (1 wt.%), the *P*_CO2_ and *α*_CO2/CH4_ were improved by 22% and 7%, respectively. A combination of GO (1 wt.%)/ZIF-8 fillers tremendously improves its *P*_CO2_; by 990% for GO/ZIF-8 (5 wt.%) and 1.124% for GO/ZIF-8 (10 wt.%). Regrettably, the MMMs lost their selectivity by 16–55% due to the non-selective filler-polymer interfacial voids. However, the hybrid MMM performances still resided close to the 2019 upper bound and showed good performance stability when tested at different feed pressure conditions.

## 1. Introduction

The energy demand of our severely industrialized world has led to an enormous amount of greenhouse gases emission. CO_2_ has been determined to be one of the most concerning factors, and its atmospheric concentration is expected to increase to 450 ppm by 2035, which might cause an increase in the global temperature by 2 °C [1]. Each 188 million tons of industrial CO_2_ emission contributes to the rise in atmospheric CO_2_ concentration by 1 ppm and the level has reached ca. 414 ppm in December 2020 [2]. The overwhelming impacts from fuel combustion heat and electricity (>40% of global CO_2_ emissions in 2014) emphasize the need for more effective CO_2_ separation from natural gas resources and CO_2_ capture in fossil fuel-based energy generation plants.

Compared to other separation technologies, membrane-based gas separation offers several benefits: low cost, better energy efficiency, relatively smaller footprint, low mechanical complexity, and continuous operation under steady-state conditions [3]. Therefore, polymeric membranes have been preferred and implemented at a large scale for CO_2_/CH_4_ separations in industries, but membrane performances are limited to the permeability–selectivity trade-off behavior [4]. Mixed matrix membranes (MMMs) introduce a new generation of composite membranes that combine the characteristics of solid or rigid filler phases (i.e., metal-organic complexes or frameworks (MOFs), carbon-based nanotubes and fillers such as graphene oxide (GO), and many more [5]), dispersed in the polymer matrix and provide a technically feasible solution to overcome the trade-off boundaries. Moreover, these membranes simultaneously overcome the limitations of both inorganic (low mechanical resistance, scale-up difficulty, high capital cost) [6] and polymeric membranes (low thermal and chemical stability, membrane plasticization) [7,8]. A good polymer-inorganic filler interaction should prevent particle agglomeration, thus enhancing the dispersion homogeneity and avoiding pore blockage, void formation, and polymer rigidification [6,9].

Zeolitic imidazolate frameworks (ZIFs), a MOF subclass, are often presented in MMMs for gas separation. ZIF is based on imidazolate (im) anionic organic ligands, tetrahedrally coordinated transition metals (M = Fe, Co, Cu, Zn) and possesses zeolite sodalite topology (SOD) [10,11]. The 145° M-im-M bridges give their tetrahedral topological networks. ZIF-8, the best known ZIF comprised of [Zn(mim)_2_]·nG (Hmim = 2-methyl imidazole, G = guest) crystallites, has shown promising properties in CO_2_ separation and capture due to its high CO_2_ adsorption capacity (up to 0.8 mmol g^−1^ at 1 bar, 25 °C [12]), owing to its inherent large pore size of 11.6 Å with a small six-membered ring pore apertures of 3.4 Å, and high surface area (up to ca. 1700 m^2^ g^−1^) [13]. Additionally, ZIF-8 adsorbs preferentially in the order of CO_2_ > CH_4_ > N_2_ [14], making it ideal for separating CO_2_ molecules (ca. 3.3 Å) from other larger kinetic diameter molecules, i.e., N_2_, CH_4_, O_2_, C_2_H_6_. As for carbon-based fillers, the GO nanosheets stood out due to their high aspect ratio with specific gas permeation pathway reducing the diffusion of larger gas molecules and simultaneously increasing the diffusion selectivity [15]. Their various functional groups, such as carboxylic acid on the edges coupled with hydroxyl and epoxy on the base planar surfaces [16], are the reactive sites for covalent functionalization and signifies GO capability to be incorporated into a MMM [17]. Additionally, the abundance of these oxy-groups could be further functionalized to elevate CO_2_ affinity and enhance the selectivity [17,18].

Several researchers have demonstrated that the combination of GO and ZIFs is made possible to synergistically benefit from the two different fillers by an in-situ synthesis of ZIF nanoparticles on the GO nanosheets. The method indicates the nucleation of ZIFs’ metal ions at the GO defective sites (which are resided by functional groups) as the first step [19,20,21]. However, due to the stacking and spontaneous curling of GO and ZIF particles’ repulsion effects, it requires a more elaborated method to ensure growth success, such as an ultrasound-assisted pre-Zn(II) doping [19]. Using a more straightforward approach, Sarfraz and Ba-Shammakh [22] physically blended GO and ZIF-301 nanoparticles at various loadings (GO, 1–5 wt.%; ZIF-301, 6–30 wt.%) into polysulfone and achieved similar synergetic effects. Their PSF/GO(1)/ZIF-301(30) MMM showed 200% CO_2_ uptake improvement (12.9 cm^3^·g^−1^) compared to the neat PSF (4.3 cm^3^·g^−1^). The MMM showed tremendous CO_2_/N_2_ selectivity improvement of 155%, directly contributed by the high CO_2_ permeability increase by 290%.

This work investigates 6FDA-DAM:DABA co-polyimide (co-PI) membranes on their potential in CO_2_ separation. Besides chemical crosslinking [23,24,25], the MMM approach has been proven to show effective CO_2_ permeability and CO_2_/CH_4_ selectivity improvements [26,27,28,29]. We studied the synergistic effect of filler addition in several possible polymer-filler combinations by varying filler types and loadings, i.e., MMMs with only ZIF-8 or GO and combining GO and ZIF-8. Gas separation performances were evaluated with an equimolar CO_2_/CH_4_ binary mixture at a constant feed pressure of 2 bar, at 25 °C. The membranes’ structural characteristics and gas transport properties are discussed accordingly.

## 2. Materials and Methods

### 2.1. Materials

For 6FDA-DAM:DABA co-PI synthesis, (4,4′-hexafluoroisopropylidene) diphthalic anhydride (6FDA, 99%), 2,4,6-trimethyl-1,3-diaminobenzene (DAM, 96%), and 3,5-diaminobenzoic acid (DABA, 98%) monomers were pre-dried at 80 °C in an oven to discard any moisture before use. DABA diamine was selected for its carboxyl groups that possess high CO_2_ affinity and thus increases CO_2_ solubility in the produced membrane matrix [24]. Additionally, the functional group also acts as reaction sites for hydrogen bonding and possible charge-transfer-complex (CTC), an intra- and intermolecular bond prominently occurs in aromatic polyimide membranes due to these electron acceptor/donor groups [26,30]. The CTC phenomenon may result in higher gas selective membranes. The solvents, 1-methyl-2-pyrrolidone (NMP, ≥99%), and tetrahydrofuran (THF, ≥99.9%) were used as received.

In the ZIF-8 synthesis, zinc nitrate hexahydrate (Zn(NO_3_)_2_·6H_2_O, ≥99.0%), 2-methylimidazole (Hmim, 99%), CHROMAPUR^®^ methanol (MeOH, 99.8%) and NMP were used. All chemicals were purchased from Sigma Aldrich (Czech Republic). The graphite powder (2–15 μm, with a purity of 99.9995%) for GO synthesis was obtained from Alfa Aesar (Germany). The sulfuric acid (98 wt.%), potassium permanganate (99.5%), phosphoric acid (85%), and hydrogen peroxide (30%) were obtained from Penta (Czech Republic).

### 2.2. Co-Polyimide and Nanomaterials Syntheses

**Figure 1:**
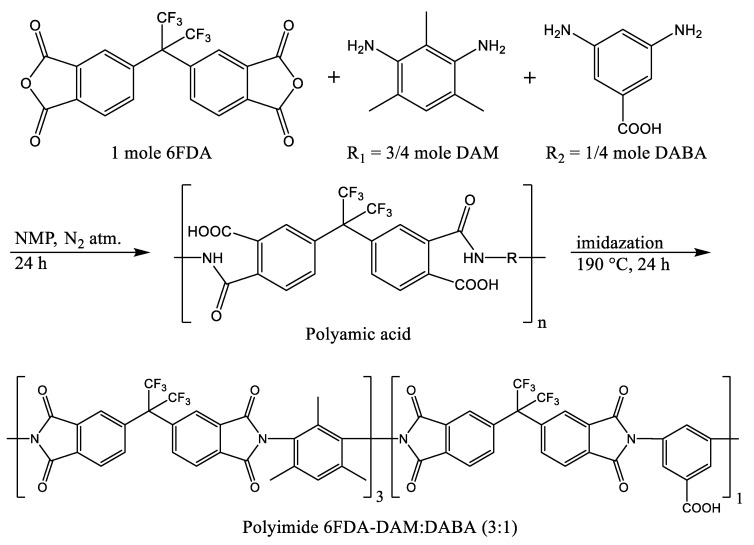
Synthesis of 6FDA-DAM:DABA co-PI. The 3:1 notation indicates the DAM to DABA diamine molar ratio.

The co-polyimide was synthesized using a two-step poly-condensation method as presented earlier [31]. Firstly, a polyamic acid (PAA) was synthesized with an equimolar amount of diamines and dianhydride monomers in NMP under a N_2_ atmosphere at room temperature. A combination of DAM and DABA diamines were used at the molar ratio of 3:1. The amount of reactants was calculated to obtain 6 g of 15 wt.% PAA solution. As the second step, thermal imidization was conducted to obtain the imidized 6FDA-DAM:DABA (3:1) polyimide (Figure 1).

ZIF-8 was synthesized by adding a solution of Zn(NO_3_)_2_·6H_2_O (1.03 g in 70 mL methanol) into the 2-methylimidazole solution (2.07 g in 70 mL methanol), and the slurry solution was stirred for 1 h. The nanoparticles were collected through two methods, described previously [32], as follows:

1. Dry method: The slurry solution was centrifuged at 20,000 rpm (20 min) and the supernatant methanol was removed and replaced with fresh methanol (30 mL), followed by an ultrasound sonication (Kraintek K-10LE, ultrasonic power 300 W at the frequency 38 kHz for 15 min) to re-disperse the nanoparticles in the fresh solvent. This procedure was repeated for 3 cycles and the final supernatant methanol was discarded. The obtained nanoparticles were dried at 90 °C overnight.
2. Wet method: At the third cycle of the dry method, the methanol was exchanged by 30 mL NMP and centrifuged at 20,000 rpm (20 min). The supernatant NMP was discarded and replaced by fresh NMP and the cycle was repeated 5 times, producing a solution of ZIF-8 nanoparticles in NMP. To determine the ZIF-8 powder concentration, 1 g of ZIF-8/NMP solution was spread onto a glass plate and kept dry in a vacuum oven at 90 °C for 24 h. The final dried weight was used to calculate the ZIF-8 concentration and determined at 0.074 g·mL^−1^. The solution was tightly sealed and kept stirred at room temperature before the MMM preparation.

Please note that the ‘dry’ ZIF-8 nanoparticles were used for characterization only, whereas the ‘wet’ ZIF-8 was used to prepare MMMs.

In the preparation of GO, the graphite was oxidized via the improved Hummers method, also called the Tour method, according to the procedure described by Jankovský et al. [33]. The graphite (3 g) was mixed with 360 mL of H_2_SO_4_ (98 wt.%) and 40 mL of H_3_PO_4_ (85 wt.%). Subsequently, 18 g of KMnO_4_ were added. The reaction mixture was stirred and then heated to 50 °C for 12 h. Afterward, the reaction mixture was quenched in ice (400 g) with 20 mL of H_2_O_2_ (30 wt.%). The formed GO was separated by centrifugation, washed several times by deionized water until neutral pH, and finally lyophilized. The resulting powder was stored in a desiccator.

### 2.3. Membrane Fabrication

In the preparation of neat membranes, 0.5 g of co-PI was dissolved in a pre-weighted solvent (THF), making a polymeric solution with a concentration of 7.0 wt.% (calculated from Equation (1)). The solution was magnetically stirred for 2 h, followed by 30 min sonication before pouring onto a casting glass and dried in a closed container of THF-saturated atmosphere for 24 h.

The dried membrane was detached by water and dried at 80 °C for 24 h before use. The procedure illustration is presented in Figure S1.

(1) $$Polymer\;conc.\;\left(wt.\%\right)=\;\frac{wt.\;of\;polymer\;\left(g\right)}{wt.\;of\;polymer\;\left(g\right)+wt.\;of\;solvent\;\left(g\right)}\;\times 100\%$$

When preparing the MMMs, the filler loadings were calculated using Equation (2). The pre-weighted fillers were first dispersed in the solvent (THF) and sonicated (Kraintek K-10LE) to disperse the nanosheets or nanoparticles before adding 1/3 of the total polymer weight for a priming step to ensure homogenous filler dispersion [31,34]. The GO and ZIF-8 MMMs were prepared with 1–4 wt.% and 5–15 wt.% loadings, respectively, in 7.0 wt.% solutions. The final GO/ZIF-8 MMMs were prepared at fixed 1 wt.% GO with various ZIF-8 loading (5–15 wt.%).

All the membranes were casted and dried as above. The illustrations for MMM preparation are presented in Figures S2–S4.

(2) $$Filler\;loading\;\left(wt.\%\right)=\;\frac{wt.\;of\;filler\;\left(g\right)}{wt.\;of\;polymer\;\left(g\right)+wt.\;of\;filler\;\left(g\right)}\;\times 100\%$$

### 2.4. Characterization

The final polyimide was characterized by a Bruker Fourier-transform infrared spectroscopy (FTIR), equipped with an IFS 66v/s to confirm that the PAA’s complete imidization was achieved. The spectral analysis was carried out from 400 to 4000 cm^−1^ wavenumbers at 4 cm^−1^ resolution. The same analysis was conducted for all membrane samples. ZIF-8′s crystallinity was determined by X-ray powder diffraction (XRD) analysis using an XRD-Diffractometer PANalytical X’Pert PRO (PANalytical Holland) using Cu-Kα radiation at a 40 kV voltage and 30 mA current.

All materials and membrane samples were imaged by a field emission scanning electron microscope (FESEM) JEOL-JSM-5600LV. The membrane samples were prepared through the freeze-fracturing method [24,35]. The membranes were immersed in liquid nitrogen for several minutes and fractured inside the liquid nitrogen for neatly fractured membrane cross-sections. Thermogravimetric analysis (TGA) was carried out using a Linseis STA 700LT where an 8–15 mg sample was placed into an alumina crucible and heated at 10 °C min^−1^ up to 700 °C under 20 mL min^−1^ N_2_ flow. The decomposition temperature (T_d_) was determined by the highest point of its first derivative of weight loss.

### 2.5. Gas Separation Measurement

Gas separation analyses were performed using a laboratory-scale permeation apparatus, with a Wicke–Kallenbach permeation cell. The set-up scheme is presented elsewhere [36]. The measurement was carried out at steady-state conditions using a CO_2_ and CH_4_ binary mixture as the feed and helium as a sweep gas. The permeate gas was analyzed using a FOCUS gas chromatograph equipped with a methanizer and a flame ionization detector (FID).

The base separation performance was performed using 50:50 vol.:vol. of CO_2_ (>99.9%, SIAD) and CH_4_ (>99.7%, Linde), at 25 °C. The gas permeabilities (reported in Barrer, 1 Barrer = 10^−10^ cm^3^(STP)·cm·cm^−2^·s^−1^·cmHg^−1^) under mixed gas conditions were calculated using Equation (3), where *y*_i_ and *x*_i_ are the molar fraction of the corresponding gas in the permeate and the feed flow, respectively. *F^s^* is the calibrated sweep flow (cm^3^(STP)·s^−1^), *l* is the membrane thickness (cm), *A* is the membrane area (cm^2^), and *P^f^* and *P^p^* are the feed and permeate side pressures (cmHg), respectively. These equations are derived from the cell mass balance, assuming the negligible cross membrane flow compared to the feed and sweep flow.

(3) $$P_{CO2}=\;\frac{y_{CO2}F^{s}l}{A\left(x_{CO2}P^{f}-y_{CO2}P^{p}\right)};\;P_{CH4}=\;\frac{y_{CH4}F^{s}l}{A\left(x_{CH4}P^{f}-y_{CH4}P^{p}\right)}$$

The separation factor of the membrane was calculated from Equation (4).

(4) $$\alpha_{CO2/CH4}=\frac{y_{CO2}/y_{CH4}}{x_{CO2}/x_{CH4}}$$

## 3. Results

### 3.1. Materials Characterizations

#### 3.1.1. 6FDA-Copolyimide and Nanoparticle Characterizations

**Figure 2:**
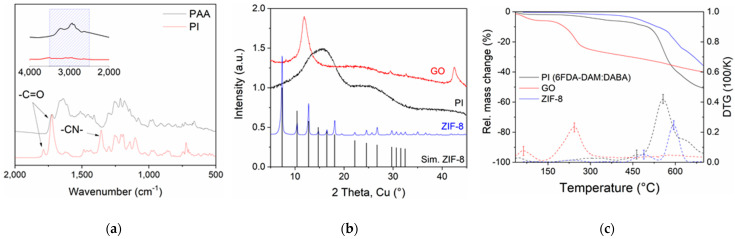
(**a**) FTIR spectra of the PAA and its imidized 6FDA-DAM:DABA (3:1) co-PI. Highlighted are their key characteristic peaks, as discussed in the text, (**b**) XRD patterns of ZIF-8 nanoparticles compared to its simulated reference peaks, polyimide and GO, and (**c**) TGA decomposition curves (straight lines) of all the materials and their first derivative (dotted lines), analyzed in N_2_.

FTIR spectra of the synthesized 6FDA-DAM:DABA (3:1) PAA and its imidized co-PI are presented in Figure 2a. A complete polyimide formation was indicated by the disappearance of amide functional group, –CONH at ~1510 cm^−1^ into imide, –NH– at ~1638 cm^−1^ [24], and the disappearance of PPA’s –COOH and –NHCO broad convoluted stretching band between 2500 and 3500 cm^−1^ [37]. Other defining co-PI peaks are the symmetric and asymmetric C = O stretching at ~1724 cm^−1^ and ~1788 cm^−1^, as well as the –CN– stretching peak at ~1358 cm^−1^. The co-PI was then used to fabricate neat flat sheet membranes and MMMs with GO (1–4 wt.%), ZIF-8 (5–15 wt.%), and their mixtures.

**Figure 3:**
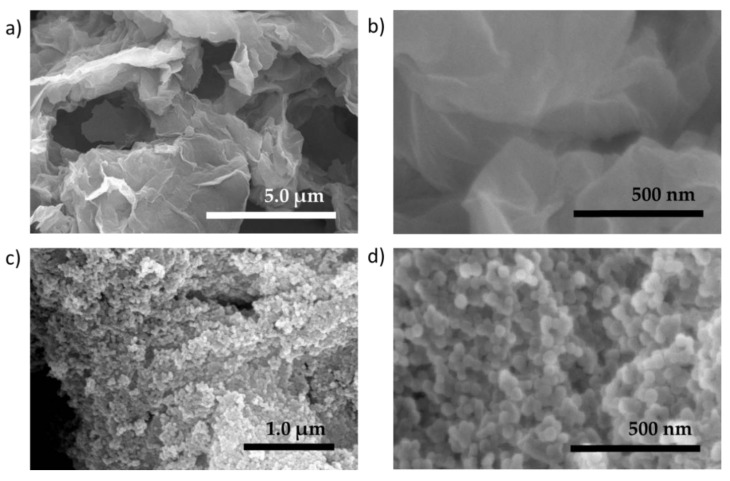
FESEM images of (**a**,**b**) graphene oxide (GO) nanosheets and (**c**,**d**) the synthesized ZIF-8 nanoparticles, in size range of ca. 37.1 ± 8.4 nm. Particle size is measured by open-source Image J.

Figure 2b shows the XRD patterns for ZIF-8, obtained at 25 °C well-consistent to ZIF-8 simulated pattern. 2*θ* values of 7.3°, 10.4°, 12.8°, 16.5° and 18.1° correspond to the crystal lattice directions of (110), (200), (211), (210), and (222), respectively. The primary peak at (110) indicates ZIF-8 face orientation and its high intensity is attributed to the stable rhombic dodecahedron shape in ZIF-8 formation, which resembles the final stage of ZIF-8 structure growth [11,34]. The FESEM images of ZIF-8 show very small nanoparticles in size range of ca. 37.1 ± 8.4 nm (see Figure 3c,d). On the other hand, GO nanosheets (FESEM images in Figure 3a,b) show a sharp 2*θ* peak at 11.8° (Figure 2b), attributed to the (001) GO direction. These values can be used to determine the nanoparticle pore opening or GO interlayer or polymer packing distances using Bragg’s law (2d_hkl_ sin *θ* = nλ; d_hkl_ = distance) [26]. As for the 6FDA-DAM:DABA (3:1) co-PI, the pristine membrane shows a broad prominent characteristic peak of an amorphous polymer with a *d*-spacing of 5.7 Å, smaller than pristine 6FDA-DAM membranes (*d*-spacing = 6.8–7.0 Å [38,39]), which will directly associate with the polymers’ smaller free volumes.

Referring TGA profile in Figure 2c, the decomposition temperature (T_d_) of neat co-PI is at ca. 557 °C, determined by the peak of the final weight loss (~41.5%) that designates the decomposition of a PI backbone. The earlier weight loss (~4.0%), initiated at ~351–490 °C, is related to the decarboxylation of DABA moieties in the 6FDA-DABA homo-polyimide, releasing CO_2_ and CO [23]. The earliest weight loss (<100 °C), which is almost negligible, is related to the evaporation of surface and near-surface trapped moisture and volatile solvent (THF, b.p. 66 °C). The following that is a near-plateau weight loss (~5.3%) up to ~350 °C associated with the deep-matrix residual removal [26]. As for ZIF-8, the analysis showed that the nanoparticles are stable up to ca. 420 °C, similar to the reported thermal stability values for nano-sized ZIF-8 in N_2_ [40]. The earlier weight loss (~1.6%) is attributed to the removing trapped moisture, guest molecules, and potentially the unreacted reactant species from the near surfaces [40,41]. In the case of GO, the initial weight loss around 90–120 °C is attributed to the dehydration of the nanosheets, followed by the main decomposition profile that peaks at ~240 °C and showing ca. 23.6% mass loss, attributed to the carboxylic decomposition, releasing CO_2_ gas [16]. The removal of hydroxyl and carboxylic groups will leave space vacancies and topological defects throughout the nanosheets planes, with smaller interlayer spacing and referred to as reduced GO (rGO).

#### 3.1.2. Membrane Characterizations

TGA was conducted to determine the decomposition profiles and effect of filler incorporation on T_d_ of membranes. FTIR spectroscopy was used to determine a possible chemical interaction between the filler and polymer, while FESEM imaged the membranes’ cross-section microstructures (thickness of 50–65 μm).

**Figure 4:**
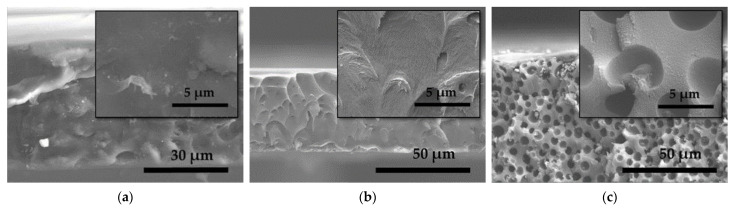
SEM images of 6FDA-DAM:DABA (3:1) co-PI with a mixture of 1 wt.% GO and (**a**) 5 wt.%, (**b**) 10 wt.% and (**c**) 15 wt.% ZIF-8 nanoparticles.

In all the GO MMMs, the T_d_ (555–557 °C, see Table 1) is similar to that of the neat membrane (557 °C); indicating GO addition brings no significant effect on the overall MMM thermal stability. Their FESEM images showed continuous and undisruptive phases as can be seen in Figure S5. Contrarily, in ZIF-8-containing MMMs, the T_d_ (553–556 °C) slightly decreases, probably due to its higher degree of polymer chain disruption upon ZIF-8 addition and the insufficient or poor interface interaction, which can be mainly observed in the FESEM images of high loading MMMs (see Figure S6, defects are highlighted in red circles). Evidently, the addition of ‘wet ZIF-8′ in the NMP solution may have contributed to the more considerable amount of deep-matrix trapped solvents (9.5–20.0% in ZIF-8-containing MMMs, see Table 1), which was removed between 100–350 °C. The value is much smaller in neat and GO MMMs. In the FESEM image of GO/ZIF-8 (15 wt.%) (Figure 4c), it can be clearly seen that the MMM possesses a good filler distribution. The round cavities surround the nanoparticles suggest that the NMP may have encapsulated the ZIF-8 nanoparticles during the preparation stage and when cured, the NMP was removed (not entirely, as evidenced in TGA), leaving space voids without filler-polymer interaction. This defective microstructure will influence the gas separation performances and be discussed accordingly in the next section.

These findings are further supported by FTIR analysis, where all membranes (GO, ZIF-8, GO/ZIF-8 MMMs) show no significant shift in the specific co-PI functional group signals; imide –NH– at ~1638 cm^−1^, symmetric and asymmetric C = O stretching at ~1724 cm^−1^ and ~1788 cm^−1^, and –CN– stretching peak at ~1358 cm^−1^, indicating there is no strong filler-polymer interaction. The spectra can be referred to in Figure S8.

### 3.2. Gas Transport Properties

#### 3.2.1. Gas Permeability and CO_2_/CH_4_ Selectivity

The thin dense 6FDA-DAM:DABA (3:1) membranes show a good CO_2_ permeability (*P*_CO2_) of 147.4 ± 6.1 Barrer and CO_2_/CH_4_ selectivity (*α*_CO2/CH4_) of 47.5 ± 4.0. The selectivity value is higher than the 6FDA-DAM:DABA (3:1) membrane reported earlier by our group [31]; nonetheless, the performances are comparable to several other studies (the values are summarized in Table 2). The synthesis procedure does not guarantee the regular distribution of DAM and DABA in polymeric chains, which leads to their random sequence distribution, hence explains the separation performance discrepancy. Despite the fact that in global the ratio of 6FDA-DAM and 6FDA-DABA sequences is 3:1, we can also expect in polymeric chain the presence of segments where the sequences of 6FDA-DAM are more cumulated and vice versa. In an extreme case, it can result in the presence of some fraction of 6FDA-DAM and 6FDA-DABA homo-polyimides in the 6FDA-DAM:DABA co-PI. From the data presented in Table 2, we may speculate that higher gas permeabilities are obtained in the 6FDA co-PI membranes with a higher fraction of 6FDA-DAM homo-polyimide or presence of longer segments with 6FDA-DAM sequences. It is known that the bulkier DAM moieties in polymers contributes to their higher free volumes, FFV (6FDA-DAM of 18–24% [39,42,43]) > 6FDA-DABA of 18.3% [44]) and bigger inter-chain distances, *d*-spacing (6FDA-DAM of 6.8 Å > 6FDA-DAM:DABA of 5.6 Å > 6FDA-DABA of 5.1 Å [38]), affected by its inefficient chain packing. This would influence the ability of small molecules to diffuse through the glassy polymer matrix. As for the lower permeability shown by 6FDA-DAM:DABA (2:1) compared to (3:2) in Table 2, it is merely caused by the higher feed pressure, which is related to the gradual saturation of permeating gases inside the polymer permanent voids, affecting the overall gas mobility through the membrane matrix rather than the competitive sorption [28].

**Figure 5:**
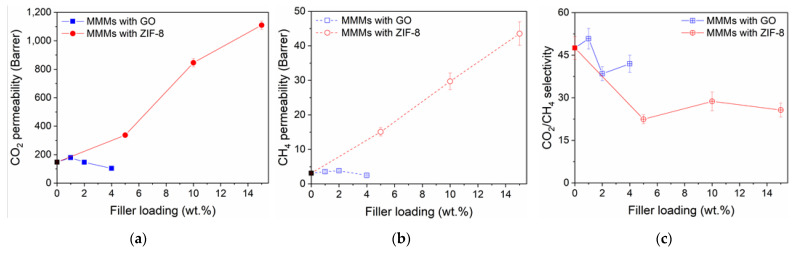
(**a**) CO_2_ permeability, (**b**) CH4 permeability and (**c**) CO_2_/CH_4_ selectivity of the pristine 6FDA-DAM:DABA (3:1) and its MMMs with GO and ZIF-8 at different loadings, tested with 50:50 vol.% of CO_2_ and CH4 binary mixture, at 2 bar and 25 °C.

**Figure 6:**
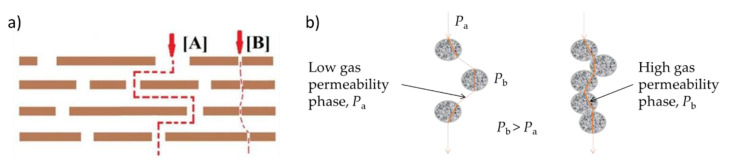
(**a**) The proposed gas transport model through GO membranes [15] and (**b**) the diffusion pathways through porous filler phases in the polymer matrix, where a discontinuous (left) or continuous (right) particle distribution is formed. The permeability in the order of *P*_b_ > *P*_a_ [5]. All images are reproduced with permissions.

It is known that a dry GO film is impermeable for all gas molecules and only water vapor permeation is allowed [45]. However, in the presence of defects in their sheet-like morphology, GO nanosheets act like a sieve that permits relatively smaller sized CO_2_ to permeate but restricts comparatively larger-sized CH_4_ molecules to pass through the pores. As expected, CO_2_ permeability in GO MMMs only increases at low loadings (1 wt.% GO MMM, *P*_CO2_ = 179.4 ± 3.6 Barrer, *α*_CO2/CH4_ = 50.8 ± 3.6) (see Figure 5a). A similar observation was reported in the highly permeable Pebax^®^1657 [46] MMMs with GO. At the higher loadings (2 and 4 wt.% GO), the membranes lost their gas permeabilities. Higher permeability would be expected at higher GO loadings, as observed in many studies [22,47,48], but we observed otherwise. This discrepancy can be explained by the two transport pathways, as presented by Ibrahim and Lin [15] and illustrated in Figure 6a. The inter-sheet pathway A comprises randomly distributed nanoscale wrinkles and inter-galleries between stacked GO sheets, which will increase the permeating gas’s tortuosity pathway and thus decrease the gas permeability. Whereas the inner-sheet pathway B constitutes of GO sheet structural defects, assumed to be aligned like straight channels, and produces a much smaller tortuosity factor for pathway B and faster gas permeation rate.

Based on these transport models, it can be concluded that in the 1 wt.% GO MMM the pathway B arrangement is primarily generated. However, at the higher GO loadings, the GO nanosheets tend to agglomerate due to GO sheets crosslinking, leading to gas diffusion channels’ blockage. The aggregated GO sheets possess a complex tortuosity [46], that reduces the CO_2_ and CH_4_ permeabilities (a similar finding is shown in Figure 5a,b). In a study using graphene sheets and PIM-1 [49], the authors simulated the MMM system and their final visualization concluded that sheets, when not agglomerated, were arranged in parallel to the polymer fragments. The arrangement constrained the polymer chain mobility, blocked and/or occupied the polymer free volumes, causing the reduced gas permeability. The phenomenon also might have occurred in our GO/6FDA-copolyimide MMMs. Meanwhile, for the other studies [22,47,48], it can be concluded that their continuous increase in gas permeability with GO loadings is caused by the non-selective voids present at GO-polymer interfaces, which were evidenced in their loss of gas pair selectivity (CO_2_/CH_4_ and CO_2_/N_2_). Overall, the reduced gas permeabilities cause the CO_2_/CH_4_ selectivity to decrease at these higher loadings (Figure 5c).

**Table 2:** Gas permeabilities (*P*) and CO_2_/CH_4_ selectivity (*α*) were obtained in this study, compared to those presented in the literature. All membranes were evaluated with 50:50 CO_2_:CH_4_ binary mixtures at 25–35 °C.

Membrane	Gas Permeability (Barrer)		CO_2_/CH_4_ Selectivity	Feed Pressure (bar)	Ref.
	CO_2_	CH_4_			
6FDA-DAM:DABA (4:1) *	320.0	-	19.7	6.9	[50]
6FDA-DAM:DABA (3:1)	199 ± 18	5.6 ± 0.3	35.9 ± 1.5	2	[31]
6FDA-DAM:DABA (3:1)	147 ± 6.1	3.1 ± 0.1	47.5 ± 4.0	2	This study
6FDA-DAM:DABA (2:1)	140.0	4.7	30.0	20	[51]
6FDA-DAM:DABA (3:2)	158.9	4.2	37.8	6.9	[52]

* CO_2_ permeance in gas permeation unit (GPU) and CO_2_/N_2_ selectivity.

As for ZIF-8 addition, the 6FDA co-PI MMMs showed a tremendous and continuous increase of CO_2_ and CH_4_ permeabilities, as shown in Figure 5a,b. The suggested gas transport model of porous filler addition; for fillers such as ZIF-8 [34,53], UiO-66 [26], HKUST-1 [54] is presented in Figure 6b, wherein a superlative case, the porous fillers will selectively enhance the permeation of smaller gas molecules and thus increase the overall MMM selectivity. Regarding the ideal case of MMM without any interface defect morphologies [9], the addition of porous selective filler should increase the gas permeability and its selectivity. Still, our observation differs from these findings (see Figure 5c). Thus, it can be suggested that there is an occurrence of non-selective interface voids, which causes the permeability increase and selectivity decrease. This phenomenon was suggested to be caused by the poor filler-polymer interaction and the polymer matrix’s inability to fully surround the large filler particles (especially agglomerates), which both lead to the non-selective permeation pathways [55]. In the highest degree of voidage, it may lead to a leaking phenomenon where the MMMs completely lost their selectivity [9]. In a very recent study, Knebel et al. [56] showed the leaking phenomenon in MMM can be resolved by using a ZIF-based porous liquid [57]. The author also ensured good filler distribution even at high particle loadings (20–50 wt.%) by using ZIF-67-IDip, a type III porous liquid in 6FDA-DAM [56,57].

In both GO- and ZIF-8-addition, we believe that the gas separation observation well agrees with their SEM images (see Figures S5 and S6), where the GO MMMs show a firm, continuous and non-disruptive phase whilst the ZIF-8 MMMs show gaps and defective interfaces around the fillers, as previously discussed.

**Figure 7:**
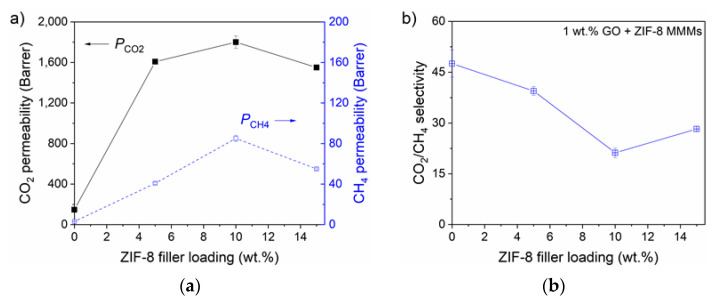
Performances of 6FDA-DAM:DABA (3:1) with 1 wt.% GO and ZIF-8 at various loadings; (**a**) CO_2_ and CH_4_ permeability and (**b**) CO_2_/CH_4_ selectivity. It was tested with 50:50 vol.% of CO_2_ and CH_4_ binary mixture, at 2 bar and 25 °C.

To further understand the potential of the 6FDA co-PI MMM, we explored a third possibility by making MMM with 1 wt.% of GO and varied ZIF-8 contents (5–15 wt.%). The obtained results of gas separation measurements are presented in Figure 7.

With the addition of 1 wt.% of GO and 5 wt.% of ZIF-8, the CO_2_ permeability was increased to 1607.2 ± 17.6 Barrer and the CH_4_ permeability to 40.8 Barrer, translated into 990% and 1216% increment respectively. The simultaneous high increase of CO_2_ and CH_4_ permeabilities is related to the interface void formation (as discussed above), indicates that the contribution of 1 wt.% GO in the binary filler MMM is marginal to improve its CO_2_/CH_4_ selectivity (reduced by 17% to a value of 39.4 ± 1.3), compared to the improved selectivity in 1 wt.% GO MMM. Further increase of ZIF-8 loading to 10 wt.% shows a similar effect where both gas permeabilities increase with a further reduction of CO_2_/CH_4_ selectivity. At the highest ZIF-8 loading (15 wt.%), the partial filler blockage and interface rigidification (other types of defective MMM morphology) can be concluded to be presence [9,26] where the gas permeability is reduced due to the blocked or stunted permeation pathways, possibly caused by the large agglomerates and polymer rigidification. The reduction instantaneously improves the overall gas selectivity by 33% (*α*_CO2/CH4_ = 28.2 ± 0.8), compared to the previous loading MMM, as presented in Figure 7b. All the gas permeation numerical data are summarized in Table S1.

#### 3.2.2. Comparison with Upper Bounds

**Figure 8:**
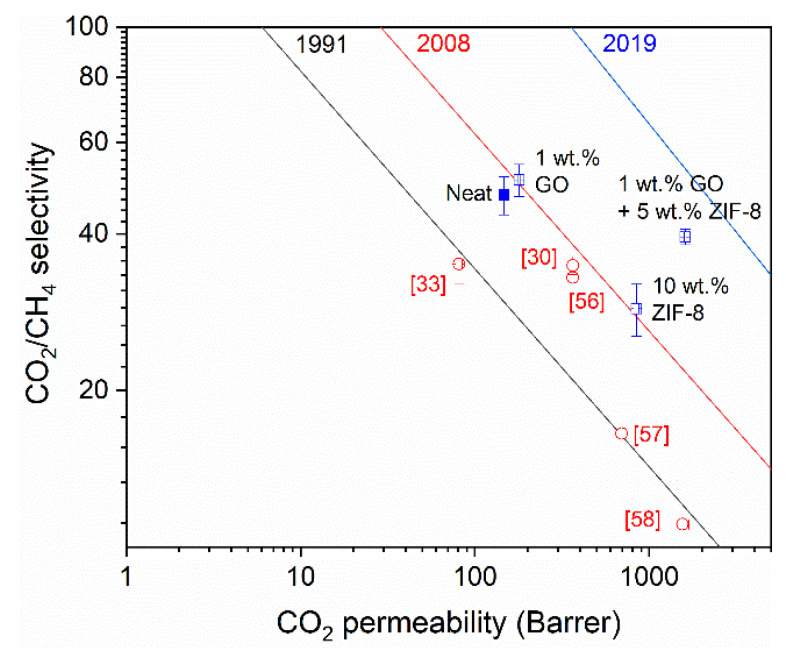
The best performing 6FDA-DAM:DABA (3:1) MMMs in this study (blue square) against CO_2_/CH_4_ upper bounds [4,58,59]. Included are several values from literature (red circles) for comparison; 6FDA-Dureen/ZIF-8 (5 wt.%) [61], 6FDA-Dureen/ZIF-8 (33.3 wt.%) [62], 6FDA-bisP/ZIF-8 (15 wt.%) [34], 6FDA-DAM:DABA (3:10 with SSZ-16 (5 wt.%) [31] and 6FDA-DAM:DABA (3:2) with DMS (20 wt.%) [60].

Figure 8 shows the performances of the best 6FDA-DAM:DABA (3:1) membranes obtained in this study, with 1 wt.% GO, 10 wt.% ZIF-8 and mixture of 1 wt.% GO and 5 wt.% ZIF-8, against the CO_2_/CH_4_ upper bounds [4,58,59]. As seen, the addition of the GO/ZIF-8 mixture pushes the 6FDA-DAM:DABA (3:1) co-PI to perform greater than the 2008 upper bound [58] and closing to the current one [59]. Additionally, for comparison, the MMM also performed better than the co-PI with other fillers such as small pore size zeolite, SSZ-16 [31] and 3D disordered mesoporous silica (DMS) [60]. Furthermore, it also performs better than other 6FDA-based polyimides with ZIF-8 [34,61,62].

#### 3.2.3. Performance at Various CO_2_ Partial Pressure

**Figure 9:**
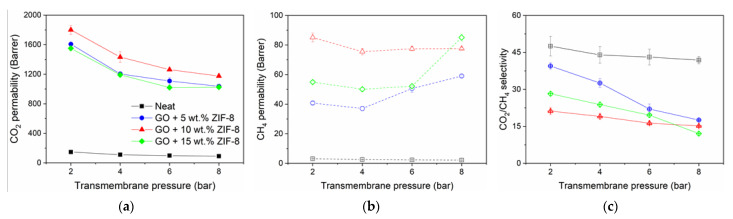
Performances of 6FDA-DAM:DABA (3:1) with 1 wt.% GO and ZIF-8 MMMs at various transmembrane pressure; (**a**) CO_2_ permeability, (**b**) CH4 permeability and (**c**) CO_2_/CH_4_ selectivity. Tested with 50:50 vol.% of CO_2_ and CH_4_ binary mixture, at 25 °C.

Figure 9 shows the gas separation performances of the neat 6FDA-DAM:DABA (3:1) and its GO/ZIF-8 MMMs when tested with 50 vol.% of CO_2_ in binary CO_2_:CH_4_ feed mixture at 2–8 bar transmembrane pressure, at 25 °C. In all samples, a continuous decrease in CO_2_ permeability is observed when the transmembrane pressure increases. The reduction is related to the well-known dual sorption model of the membrane and has been presented countlessly over the years [23,28,63,64]. When the feed pressure is increased, the polymer’s CO_2_ solubility rises, leading to polymer free volume saturation. The CO_2_-saturated polymer leads to a reduced diffusion due to reduced gas transport driving force in the membrane matrix and consequently affects the permeability. Interestingly, when comparing CO_2_ permeability reductions between 2 and 8 bar of the neat membrane (–38%) to the MMMs (−34–36%), a similar reduction percentage is presented (Figure 9a). This could be explained by the fact that the membranes possess a high degree of interface defects (evidenced by its gas permeation behavior and FESEM images), and the defects greatly influence the overall permeation, where the gases more likely to only permeate through the voids rather than selectively through the fillers. This prevents a precise observation of the positive effects of ZIF-8 addition in the MMM. Several studies had shown that several MMMs with porous fillers (e.g., SSZ-16 in 6FDA-DAM:DABA [31], UiO-66 in 6FDA-DAM [26]), when not defective, demonstrated lesser CO_2_ permeability reduction at high pressure, due to the presence of ‘strong’ filler-polymer interface interaction.

For the pressure dependence of the CH_4_ permeability (Figure 9b) over the measured pressure range, a similar case applies with regards to the continuous CH_4_ permeability reduction (as observed in the neat membrane). However, at 4–8 bar, all the MMMs showed increased permeability and it is more prominent in 5 wt.% and 15 wt.% ZIF-8 MMMs by 59% and 70%, respectively, whereas it is only 3% increase for 10 wt.% ZIF-8 MMM. As already known, the dissolved CO_2_ promotes chain mobility (which can be explained by dynamic swelling of glassy polymer matrices upon CO_2_ exposure [65]). It increases the polymer free volume pathways, causing the lower permeating CH_4_ to permeate faster [39,63]. This also suggests that the effect of chain mobility induced by absorbed CO_2_ is more prominent in the readily disrupted polymer chains in MMMs and the absence of good filler-polymer interactions. The CH_4_ permeability increase directly influences the higher CO_2_/CH_4_ selectivity reductions in the said membranes (−59% in 5 wt.% ZIF-8 MMM and –70% in 15 wt.% MMM), compared to the neat (−12%) and 10 wt.% MMM (−28%) (Figure 9c). We can safely conclude that MMMs with 5 wt.% and 15 wt.% are more defective with substantial interface voids than the 10 wt.% MMM. The permeation numerical data are presented in Table S2.

## 4. Conclusions

In this study, we presented a high-performance 6FDA-copolyimide for CO_2_/CH_4_ separation. This polymer’s gas transport properties were investigated and further enhanced with the incorporation of GO nanosheets, ZIF-8 nanoparticles, and a mixture of the two fillers. GO addition presented both CO_2_ permeability and CO_2_/CH_4_ selectivity improvement at low loading, whereas the ZIF-8 incorporation resulted in tremendous permeability improvement at the expense of its selectivity. The findings indicate that the MMMs consist of interfacial defects that act as non-selective gas permeation pathways. With the use of GO/ZIF-8 filler mixture, similar shortcomings were encountered. Nevertheless, the MMM performed close to the 2019 CO_2_/CH_4_ performance upper bound and showed good performance stability when tested at different feed pressure. Based on these outcomes, it further emphasizes the importance of non-defective MMM morphologies for small molecule separation and strengthening our knowledge in the MMM field.

## Figures and Tables

**Table 1:** The decomposition temperatures (T_d_) of the 6FDA-DAM:DABA (3:1) and its respective MMMs. Their decomposition profiles and weight loss first derivative curves are presented in Figure S7.

Membrane	Decomposition Percentage (%)			T_d_ (°C)
	100-350 °C	351–490 °C	491–700 °C	
6FDA-DAM:DABA (3:1)	5.3	4.0	41.5	557
MMM GO 1 wt.%	5.6	3.0	31.7	555
GO 2 wt.%	8.1	3.7	34.4	557
GO 4 wt.%	9.3	3.9	45.3	557
MMM ZIF-8 5 wt.%	9.6	11.3	34.4	556
ZIF-8 10 wt.%	15.9	6.6	31.7	555
ZIF-8 15 wt.%	20.0	7.7	36.8	553
MMM GO (1 wt.%)/ZIF-8 5 wt.%	11.2	11.0	31.9	555
GO (1 wt.%)/ZIF-8 10 wt.%	15.8	6.6	31.7	554
GO (1 wt.%)/ZIF-8 15 wt.%	13.8	8.2	36.4	553

## Data Availability

Not applicable.

## Supplementary Materials

The following are available online at https://www.mdpi.com/2079-4991/11/3/668/s1. Figure S1. The illustration of preparation procedure of co-PI membranes; Figure S2. The illustration of preparation procedure of co-PI with GO MMMs; Figure S3. The illustration of preparation procedure of ZIF-8/co-PI MMMs; Figure S4. The illustration of preparation procedure of ZIF-8/GO/co-PI MMMs; Figure S5. FESEM images of 6FDA-DAM:DABA (3:1) with (a) 1 wt.%, (b) 2 wt.% and (c) 4 wt.% GO nanosheets; Figure S6. FESEM images of 6FDA-DAM:DABA (3:1) with (a,b) 5 wt.%, (c,d) 10 wt.% and (e,f) 15 wt.% ZIF-8 nanoparticles. Red circles highlight the defective interfaces, a result of poor filer-polymer interface interaction; Figure S7. Thermal decomposition profiles of 6FDA-DAM:DABA (3:1) and its MMMs with (a) GO, (b) ZIF-8, and (c) GO and ZIF-8 mixtures. Included are their weight loss first derivatives; Figure S8. FTIR spectra of 6FDA-DAM:DABA (3:1) and its MMMs with (a) GO, (b) ZIF-8, and (c) GO and ZIF-8; Table S1. Gas permeabilities (*P*) and CO_2_/CH_4_ selectivity (*α*) of neat 6FDA-DAM:DABA (3:1) and its respective MMMs with GO nanosheets, ZIF-8 nanoparticles, and GO/ZIF-8 mixtures. All membranes were evaluated with 50:50 CO_2_:CH_4_ binary mixtures at 25 °C, at a feed pressure of 2 bar; Table S2. Gas permeabilities (*P*) and CO_2_/CH_4_ selectivity (*α*) of neat 6FDA-DAM:DABA (3:1) and GO/ZIF-8 MMMs, tested at different feed pressure (2, 4, 6, 8 bar). All membranes were evaluated with 50:50 CO_2_:CH_4_ binary mixtures at 25 °C.
