# Supplementary material for: 6FDA-DAM:DABA Co-Polyimide Mixed Matrix Membranes with GO and ZIF-8 Mixtures for Effective CO2/CH4 Separation
Source: Nanomaterials (Basel). 2021 Mar 8;11(3):668. doi: 10.3390/nano11030668 (PMC7999237; doi:10.3390/nano11030668)
Supplement: Supplementary file 1 [file nanomaterials-11-00668-s001.pdf]

nanomaterials
MDPI

Supporting Information

6FDA-DAM:DABA co-polyimide mixed matrix membranes with ZIF-8 and GO mixtures for effective CO_2/CH_4 separation

Anand Jain [1,2], Mohd Zamidi Ahmad [1,3,*], Audrey Linkès [1], Violeta Martin-Gil [1], Roberto Castro-Muñoz [1,4], Werner Hintz [2], Pavel Izak [5], Zdeněk Sofer [6] and Vlastimil Fila [1,*]

1 Department of Inorganic Technology, University of Chemistry and Technology Prague, Technická 5, 166 28 Prague 6, Czech Republic; A.L. (ady.jain92@gmail.com), V.M.G. (violeta.m.gil@gmail.com)
2 Faculty of Process and Systems Engineering, Otto-von-Guericke-University, Magdeburg Universitätsplatz 2, Magdeburg-39106, Germany; A.J. (ady.jain92@gmail.com), W.H. (werner.hintz@ovgu.de)
3 Organic Materials Innovation Center (OMIC), Department of Chemistry, University of Manchester, Oxford Road, Manchester M13 9PL, UK
4 Tecnologico de Monterrey, Campus Toluca, Avenida Eduardo Monroy Cárdenas 2000 San Antonio Buenavista, 50110 Toluca de Lerdo, Mexico; R.C.M. (food.biotechnology88@gmail.com; castromr@tec.mx)
5 Department of Membrane Separation Processes, Institute of Chemical Process Fundamentals of the CAS, v. v. i., Rozvojova 2/135, 165 02 Prague 6 – Suchdol, Czech Republic; P.I. (izak@icpf.cas.cz)
6 Department of Inorganic Chemistry, University of Chemistry and Technology Prague, Technická 5, 166 28 Prague 6, Czech Republic; Z.S. (Zdenek.Sofer@vscht.cz)
* Correspondence: V.F. (Vlastimil.Fila@vscht.cz); M.Z.A (mohdzamidi.ahmad@manchester.ac.uk)

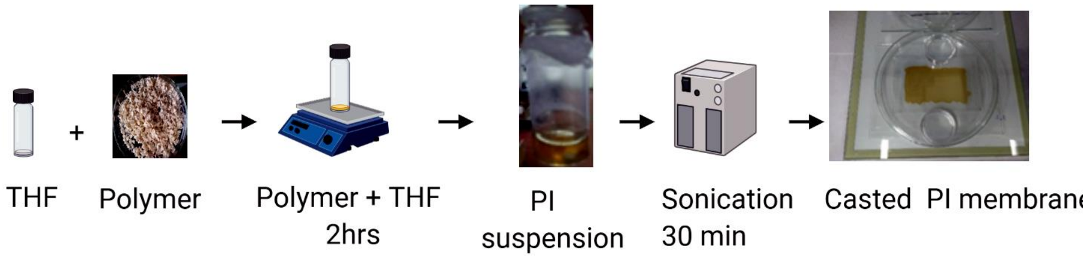

Figure S1. The illustration of preparation procedure of co-polyimide (co-PI) membranes.

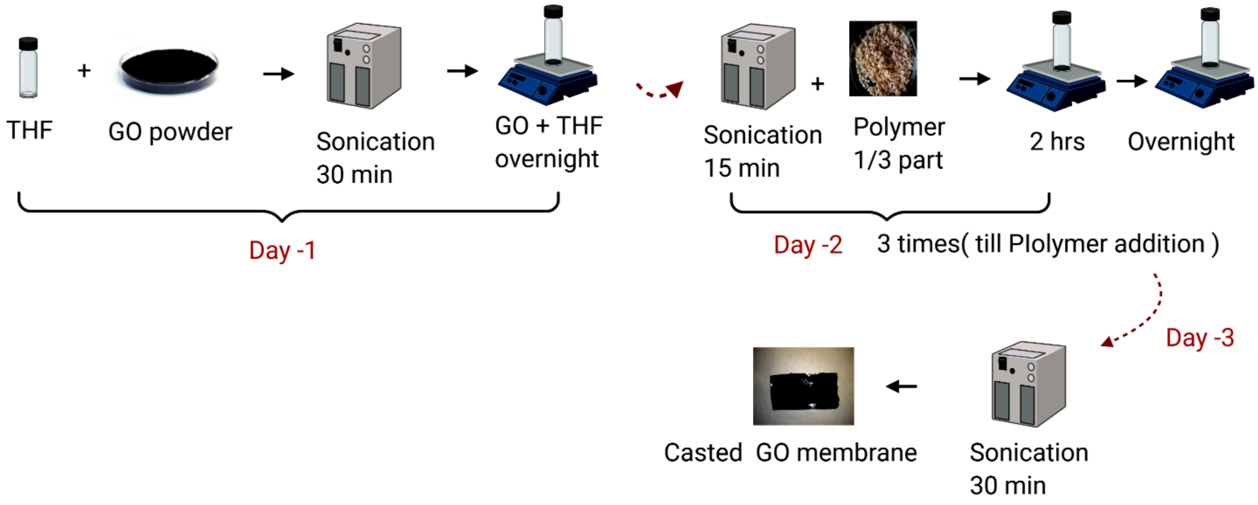

Figure S2. The illustration of preparation procedure of co-PI with GO MMMs.

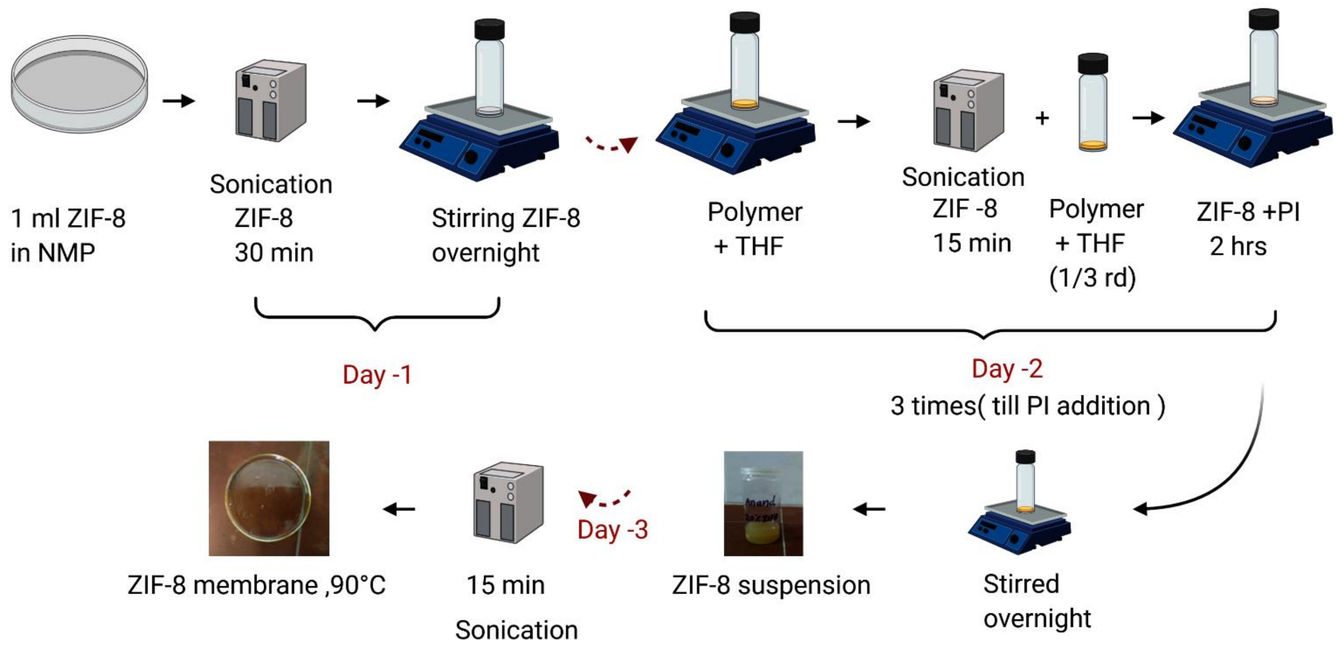

Figure S3. The illustration of preparation procedure of ZIF-8/co-PI MMMs.

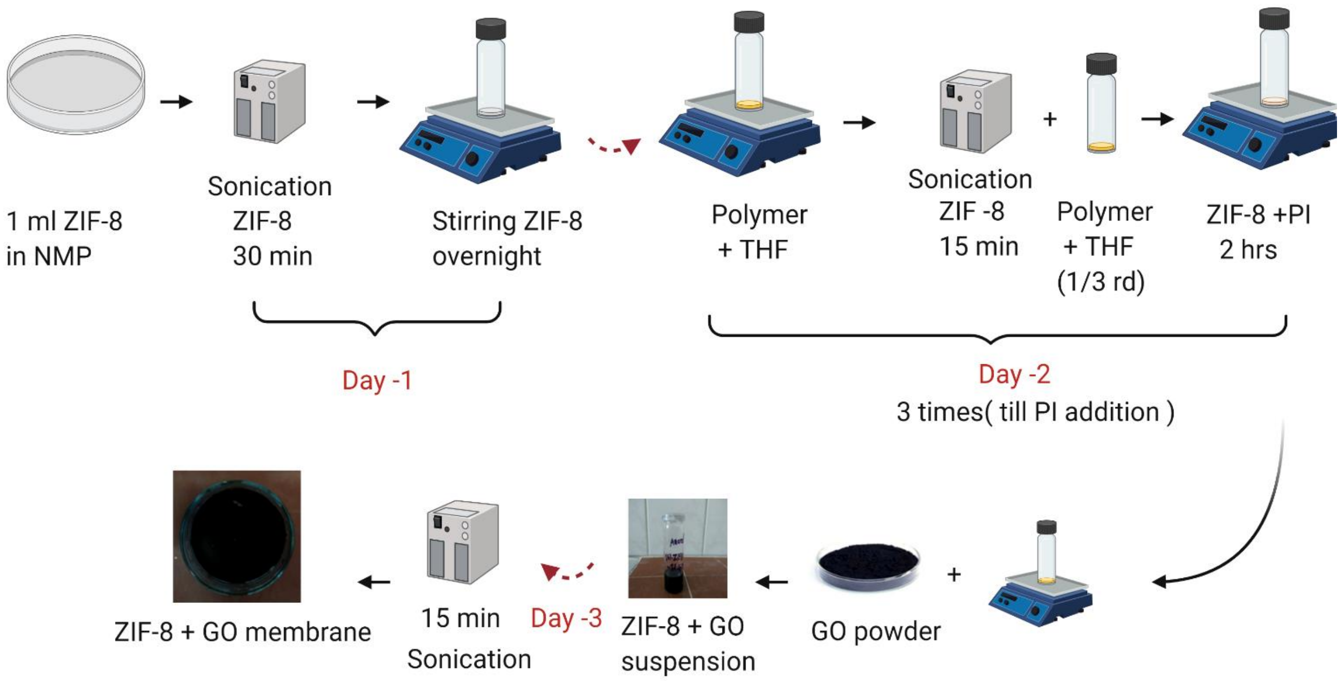

Figure S4. The illustration of preparation procedure of ZIF-8/GO/co-PI MMMs.

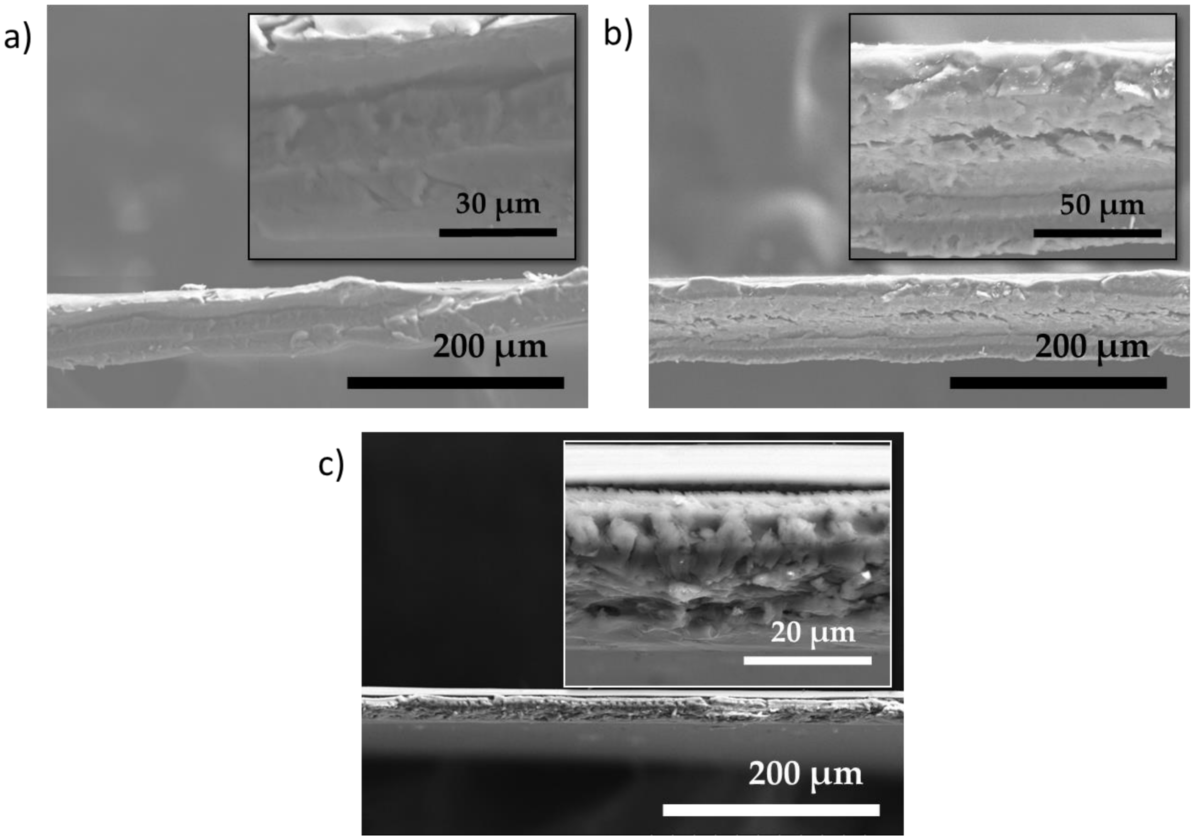

Figure S5. FESEM images of 6FDA-DAM:DABA (3:1) with (a) 1 wt.%, (b) 2 wt.% and (c) 4 wt.% GO nanosheets.

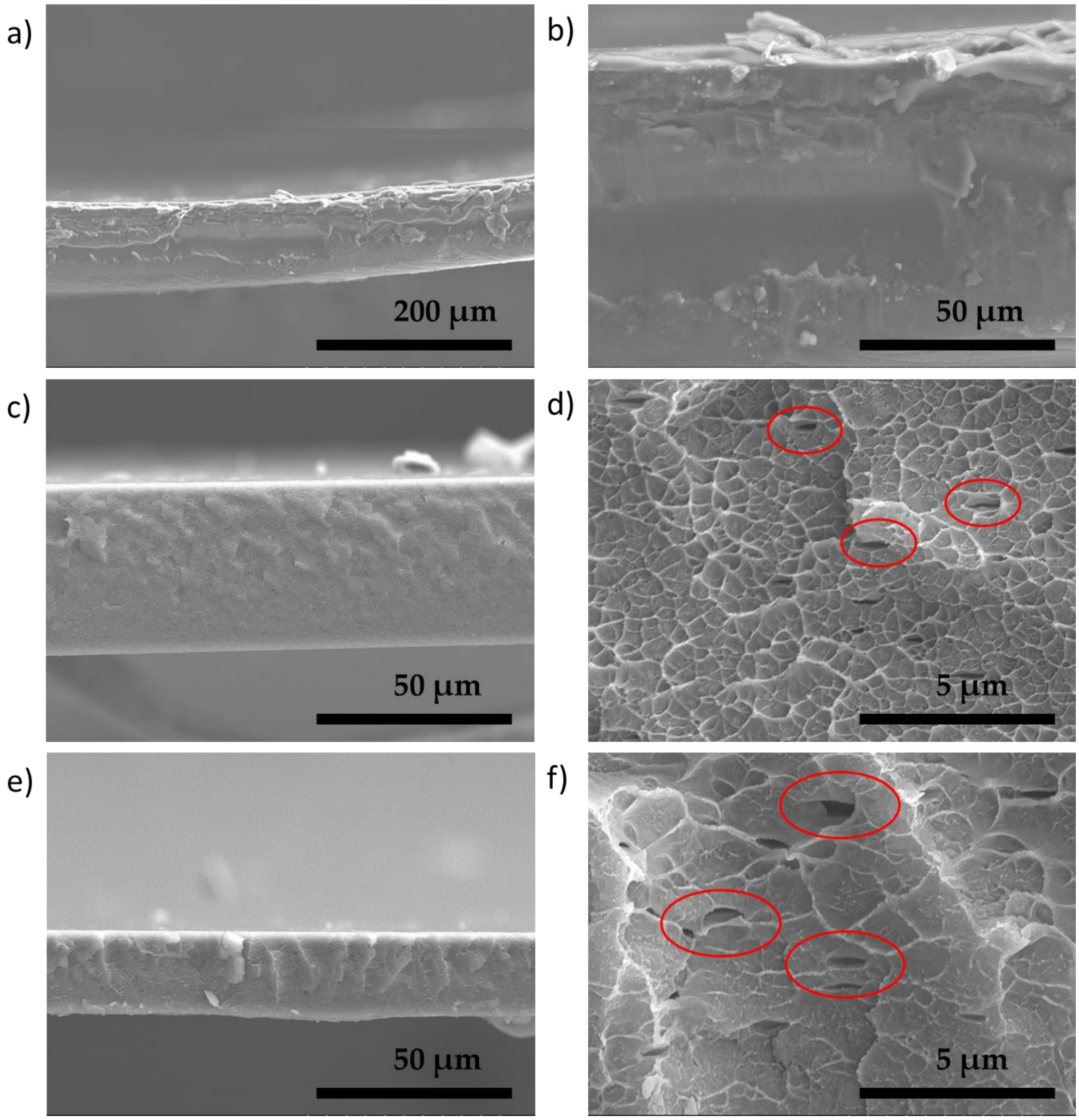

Figure S6. FESEM images of 6FDA-DAM:DABA (3:1) with (a-b) 5 wt.%, (c-d) 10 wt.% and (e-f) 15 wt.% ZIF-8 nanoparticles. Red circles highlight the defective interfaces, a result of poor filer-polymer interface interactions.

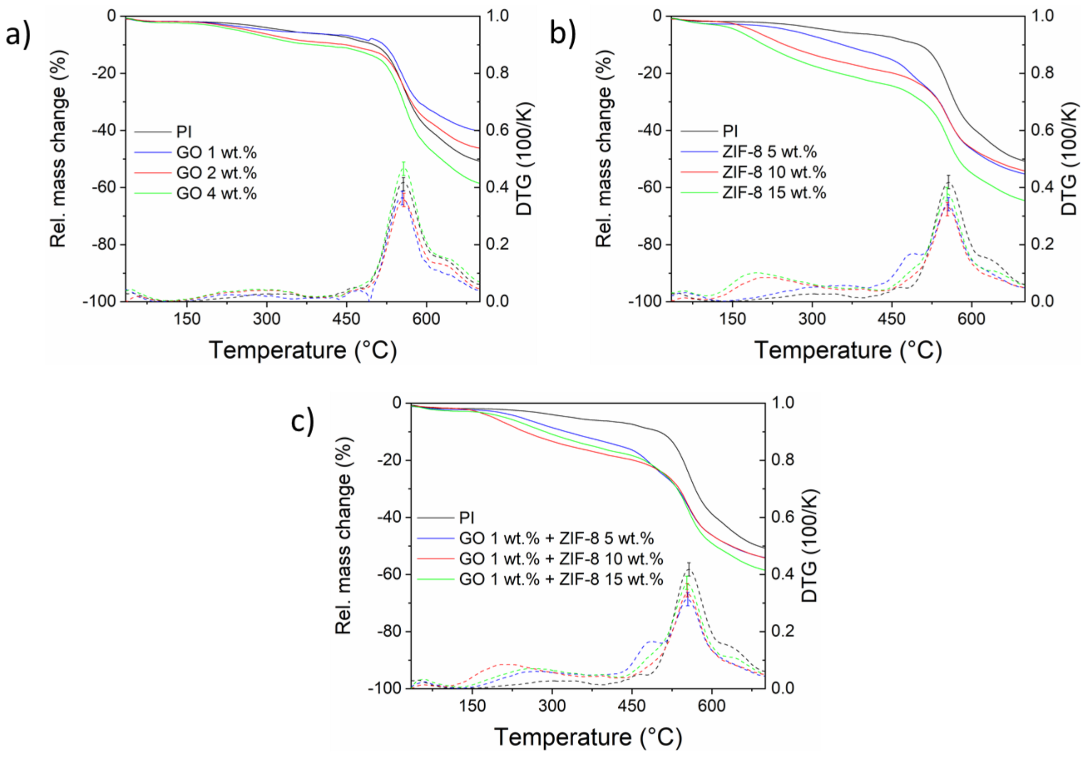

Figure S7. Thermal decomposition profiles of 6FDA-DAM:DABA (3:1) and its MMMs with (a) GO, (b) ZIF-8, and (c) GO and ZIF-8 mixtures. Included are their weight loss first derivatives.

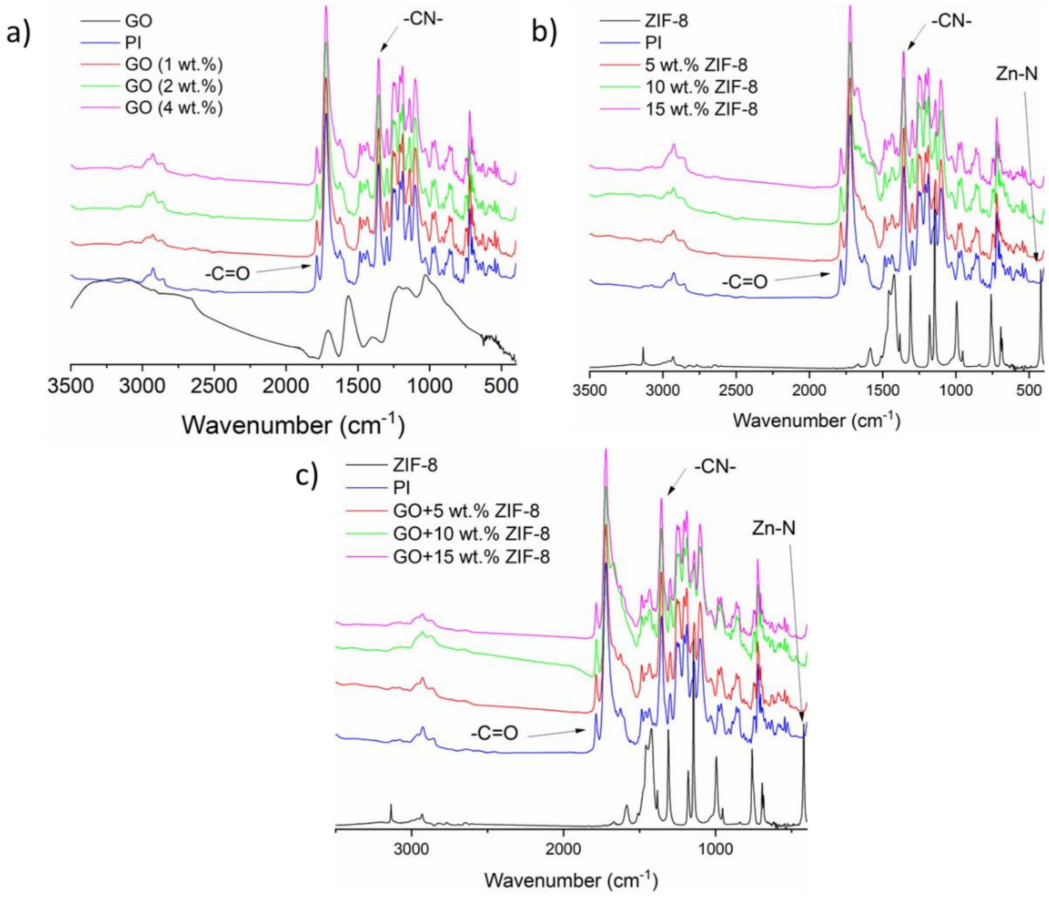

Figure S8. FTIR spectra of 6FDA-DAM:DABA (3:1) and its MMMs with (a) GO, (b) ZIF-8, and (c) GO and ZIF-8.

Table S1. Gas permeabilities (*P*) and CO_2/CH_4 selectivity (α) of neat 6FDA-DAM:DABA (3:1) and its respective MMMs with GO nanosheets, ZIF-8 nanoparticles, and GO/ZIF-8 mixtures. All membranes were evaluated with 50:50 CO_2:CH_4 binary mixtures at 25 °C, at a feed pressure of 2 bar.

Membrane	Gas permeability (Barrer)		CO_2/CH_4 selectivity
	CO_2	CH_4	
6FDA-DAM:DABA (3:1)	147 ± 6.1	3.1 ± 0.1	47.5 ± 4.0
GO MMMs			
1 wt.% GO	179 ± 3.6	3.5 ± 02	50.8 ± 3.6
2 wt.% GO	147 ± 3.5	3.8 ± 0.2	38.5 ± 2.5
4 wt.% GO	104 ± 3.9	2.5 ± 0.2	42.0 ± 3.0
ZIF-8 MMMs			
5 wt.% ZIF-8	337 ± 7.2	15.1 ± 1.2	22.4 ± 1.6
10 wt.% ZIF-8	846 ± 28.7	29.7 ± 2.4	28.7 ± 3.3
15 wt.% ZIF-8	1,109.8 ± 29.5	43.5 ± 3.4	25.7 ± 2.5
GO + ZIF-8 MMMs			
1 wt.% GO + 5 wt.% ZIF-8	1,607 ± 17.6	40.8 ± 1.0	39.4 ± 1.3
1 wt.% GO + 10 wt.% ZIF-8	1,800 ± 61.8	85.1 ± 3.0	21.2 ± 1.4
1 wt.% GO + 15 wt.% ZIF-8	1,550 ± 25.1	54.9 ± 0.8	28.2 ± 0.8

Table S2. Gas permeabilities (*P*) and CO_2/CH_4 selectivity (α) of neat 6FDA-DAM:DABA (3:1) and GO/ZIF-8 MMMs, tested at different feed pressure (2, 4, 6, 8 Bar). All membranes were evaluated with 50:50 CO_2:CH_4 binary mixtures at 25 °C.

Membrane	Pressure (bar)	Gas permeability (Barrer)		CO_2/CH_4 selectivity
		CO_2	CH_4	
6FDA-DAM:DABA (3:1)				
	2	147 ± 6.1	3.1 ± 0.1	47.5 ± 4.0
	4	111 ± 4.0	2.5 ± 0.1	44.0 ± 3.4
	6	98 ± 3.5	2.3 ± 0.1	43.1 ± 3.2
	8	91 ± 1.9	2.2 ± 0.1	41.9 ± 1.5
1 wt.% GO + 5 wt.% ZIF-8				
	2	1,607 ± 17.6	40.8 ± 1.0	39.4 ± 1.3
	4	1,205 ± 21.0	37.1 ± 1.5	32.6 ± 1.8
	6	1,108 ± 44.6	50.6 ± 2.5	22.0 ± 2.0
	8	1,036 ± 5.3	59.0 ± 0.8	17.6 ± 0.3
1 wt.% GO + 10 wt.% ZIF-8				
	2	1,800 ± 61.8	85.1 ± 3.0	21.2 ± 1.4
	4	1,432 ± 74.3	75.5 ± 2.1	19.0 ± 1.3
	6	1,262 ± 9.1	77.5 ± 1.4	16.3 ± 0.4
	8	1,176 ± 14.8	77.6 ± 0.8	15.2 ± 0.3
1 wt.% GO + 15 wt.% ZIF-8				
	2	1,550 ± 25.1	54.9 ± 0.8	28.2 ± 0.8
	4	1,192 ± 31.7	50.1 ± 1.2	23.8 ± 1.2
	6	1,021 ± 4.5	52.2 ± 0.4	19.6 ± 0.1
	8	1,025 ± 12.2	85.1 ± 2.2	12.1 ± 0.4
